# Common Garden Experiment Reveals Genetic Control of Phenotypic Divergence between Swamp Sparrow Subspecies That Lack Divergence in Neutral Genotypes

**DOI:** 10.1371/journal.pone.0010229

**Published:** 2010-04-19

**Authors:** Barbara Ballentine, Russell Greenberg

**Affiliations:** 1 Smithsonian Migratory Bird Center, National Zoological Park, University of West Georgia, Carrollton, Georgia, United States of America; 2 Smithsonian Migratory Bird Center, National Zoological Park, Washington, D. C., United States of America; Zoological Society of London, United Kingdom

## Abstract

**Background:**

Adaptive divergence between populations in the face of strong selection on key traits can lead to morphological divergence between populations without concomitant divergence in neutral DNA. Thus, the practice of identifying genetically distinct populations based on divergence in neutral DNA may lead to a taxonomy that ignores evolutionarily important, rapidly evolving, locally-adapted populations. Providing evidence for a genetic basis of morphological divergence between rapidly evolving populations that lack divergence in selectively neutral DNA will not only inform conservation efforts but also provide insight into the mechanisms of the early processes of speciation. The coastal plain swamp sparrow, a recent colonist of tidal marsh habitat, differs from conspecific populations in a variety of phenotypic traits yet remains undifferentiated in neutral DNA.

**Methods and Principal Findings:**

Here we use an experimental approach to demonstrate that phenotypic divergence between ecologically separated populations of swamp sparrows is the result of local adaptation despite the lack of divergence in neutral DNA. We find that morphological (bill size and plumage coloration) and life history (reproductive effort) differences observed between wild populations were maintained in laboratory raised individuals suggesting genetic divergence of fitness related traits.

**Conclusions and Significance:**

Our results support the hypothesis that phenotypic divergence in swamps sparrows is the result of genetic differentiation, and demonstrate that adaptive traits have evolved more rapidly than neutral DNA in these ecologically divergent populations that may be in the early stages of speciation. Thus, identifying evolutionarily important populations based on divergence in selectively neutral DNA could miss an important level of biodiversity and mislead conservation efforts.

## Introduction

Understanding how populations adapt to local ecological conditions is not only a central theme of evolutionary biology [1], [2], but also has important implications for conservation [3], [4]. The definition of evolutionary important subspecific taxa continues to be debated by conservation biologists [5], [6], [7], [8] and is of particular importance to conservation policy [9], considering, for example, that a third of the bird taxa on the US endangered species list are subspecies. In the past several decades, systematic biology has transitioned from characterizing relationships between taxonomic groups based on phenotypic characters to using molecular genetic markers such as mitochondrial DNA [10] but see [11]. The reliance on molecular genetic markers, assumed to be selectively neutral, is attractive, because biologists can seemingly objectively define taxonomic groups as a group of individuals that share a unique common ancestry of alleles (i.e. reciprocal monophyly) [7], [12] or show a quantifiable level of base-pair substitutions.

An increasing number of studies, however, report an absence of differentiation between populations when using molecular genetic markers in taxa that exhibit significant morphological divergence sufficient to be classified as subspecies or even species [13]. Differences in local environmental conditions may result in spatially varying selection, which can lead to rapid local adaptation in the absence of divergence in molecular markers [14], [15]. Thus, the reliance on divergence of traditional molecular markers to identify subspecies will result in locally adapted populations being underrepresented in the taxonomy, presenting a challenge to identify vulnerable populations as evolutionary significant units and misleading conservation efforts [10]. However, we can conclude that geographic variation reflects local adaptation only when a genetic basis for the variation is established and phenotypically plastic response to environmental differences can be ruled out [16], [17]. Therefore we use an experimental approach to directly test whether phenotypic variation in populations of swamp sparrows (*Melospiza georgiana*) is an adaptive or plastic response to divergent selection. By raising individuals from different environments in a common laboratory environment (common garden) we can differentiate between adaptive and plastic responses to divergent environments. If phenotypic divergence is maintained in experimental populations, then we can conclude that differences are due to underlying genetic divergence.

Swamp sparrows provide an excellent system in which to test the hypothesis that geographic variation results from genetically-based adaptation to a recent ecological shift from inland freshwater marshes to tidal salt marshes. North American tidal marshes are primarily post-glacial geologic features characterized by tidal flooding, high salinity, and a biotic community specialized for these conditions [18]. Coastal plain swamp sparrows (*M. g. nigrescens*, coastal) are recent colonists of tidal marshes of the mid-Atlantic estuaries and have larger bills and have both grayer and blacker plumage than populations found in inland fresh water marshes (*M. g. georgiana*, *M. g. ericrypta*, inland) [19], [20]. Furthermore, like other tidal marsh specialists, coastal plain swamp sparrows have significantly longer breeding seasons than interior populations at the same latitude (110 versus 85 days) [21]. The concordance of morphological and behavioral divergence across Emberizid sparrow taxa of North American salt marshes suggests that these features are habitat-specific adaptations [1], [20].

Despite differences in morphology and life history, there is no divergence between inland and coastal populations in a variety of molecular genetic markers that are presumably selectively neutral; allozymes [22], mitochondrial DNA [23], and microsatellites (R. Fleischer, unpub. data), possibly due to the recent separation of these populations. In this study, we use a common garden experiment to determine if morphological and life historical differences between inland and coastal populations of swamp sparrows remain when individuals are reared under identical environmental conditions.

Although some success has been reported in searching for candidate genes whose expression effects adaptive traits in birds such as bill morphology [24] and plumage [25], this approach depends upon locating specific genetic loci and failure to find divergence in candidate genes cannot rule out the complete range of genetic mechanisms underlying differentiation in these traits. Therefore we use an experimental approach to directly test whether phenotypic variation in populations of swamp sparrows (*Melospiza georgiana*) is an adaptive or plastic response to divergent selection. By raising individuals from different environments in a common laboratory environment (common garden) we can differentiate between adaptive and plastic responses to divergent environments. If phenotypic divergence is maintained in experimental populations, then we can conclude that differences are due to underlying genetic divergence.

## Results

As observed in wild populations, we found that experimental adults from coastal populations had significantly larger bills than experimental adults from inland populations (Mean bill volume ± SD, coastal  = 213.2±5.2 inland  = 174.1±3.1, t = −6.48, p<0.0001, n = 34, table 1). We found that differences in bill size between populations of experimental birds (coastal vs. inland) persisted throughout development with significant differences in bill volume appearing as early as 12 days of age (Mean bill volume ± SD, coastal  = 106.9±15.3 inland  = 91.0±8.5, t = −4.14, p = 0.0001, n = 34). Bill volume measured at fledging and after maturation was significantly correlated within individuals (r = 0.53, n = 34, p = 0.001), suggesting bill size is a stable trait. Similar to wild populations [19], there were no differences between populations of experimental birds in three measures of body size of adults (mass, tarsus, wing length, table 1).

**Table 1:** Comparison of morphological variables.

	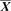 ± SE INLAND	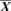 ± SE COASTAL	t-value
Mass (g)	22.7±0.8	21.8±0.9	0.82
Wing (mm)	59.8±0.5	59.3±0.5	0.79
Tarsus (mm)	22.5±0.2	22.3±0.3	0.73
Bill Length (mm)	7.7±0.1	8.3±0.1	−4.23***
Bill Width (mm)	4.2±0.1	4.6±0.1	−5.48****
Bill Depth (mm)	5.3±0.1	5.5±0.1	−2.39*
Bill Vol. (mm^3^)	174.1±3.1	213.2±5.2	−6.48****
Chestnut (%)	65±4	12±2	11.76****
Black back (%)	22±2	34±2	−4.56****
Black head (%)	26±3	59±3	−7.64****
Flank (slope)	0.037±0.002	0.027±0.002	3.42**
Crown Class (1–4)	3.3±0.2	2.7±0.3	Mann Whitney U = 177

Independent t-tests reveal differences across populations for bill and plumage variables but not body size variables (Inland n = 17, Coastal n = 17). Bill volume is a composite measure multiplying bill length, bill width and bill height.

*P<0.05,

**P<0.01,

***P<0.001,

****P<0.0001.

In experimental adults, we found significant differences (coastal vs. inland) in plumage coloration. Coastal adults had significantly more black plumage (non-breeding) on the head, back and eye line (table 1), and flank plumage was significantly less rusty (table 1). Breeding plumage in males with fully or almost fully developed crown patches were further compared (crown classification of a 3–4; n = 9 from each population). We found no difference in the length of the rusty crown patch (t = −0.24, p = 0.81). However, coastal males had more black on the forehead patch than inland males (t = −5.35, p<0.0001). Furthermore, only one inland male had more than 50% black feathers on the nape, while 10 coastal males had napes with greater than 50% black.

Previous studies have found that 100% of specimens collected in the wild could be correctly assigned to subspecies using a Discriminant Function Analysis (DFA) based on bill size and plumage characteristics [19]. For all of the laboratory-raised birds a DFA based on morphological variables was highly significant (Wilk's λ = 0.12, P<0.001) and correctly identified 100% of the individuals in a post-hoc classification based on a prior expectation of 50% correct classification by chance alone. Four variables were included in the discriminant function: Percent chestnut in eye-line (canonical coefficient  = −0.57), % black in back (0.50), bill volume (0.61), and flank color slope (−0.32). The cross-validation analysis based on bill volume and percent chestnut was highly significant (Wilk's λ = 0.09, P<0.001) and resulted in 100% correct classification.

**Figure 1:**
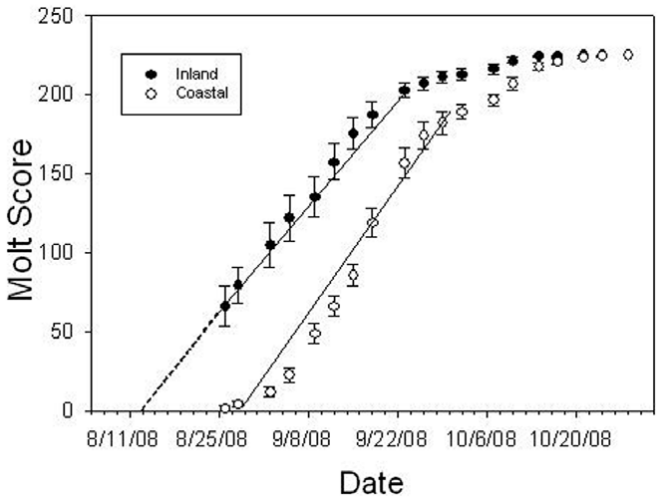
Progression of molt for experimental populations (n = 16 inland, n = 16 coastal). Molt initiation for inland adults is estimated from linear regression of molt score on date for data collected from 8/25 to 9/22.

**Figure 2:**
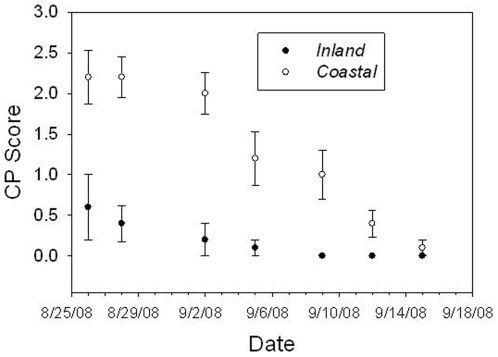
Regression of cloacal protuberance for 9 experimental males from each population. All experimental males from both populations had maximum CP scores (3) during the normal breeding season between May and July.

The timing of molt was also significantly different between experimental populations (table 2, fig. 1). Consistent with the longer breeding season observed in wild populations of coastal swamp sparrows, the experimental coastal populations initiated molt later than inland populations but progressed through molt more quickly than inland populations (table 2, fig. 1). We also found that coastal males were in breeding condition significantly later than inland males (table 2, fig. 2).

**Table 2:** Results of separate mixed procedure models on the differences between populations of experimental adults in molt scores and in reproductive status of males (cloacal protuberance score).

Effect	DF (Num) Molt Score	DF (Den) Molt Score	F-value Molt Score	DF (Num) CP Score	DF (Den) CP Score	F-value CP Score
Population	1	30	44.65***	1	18	22.13**
Date	18	540	432.53***	9	155	22.72***
Population X date	18	540	30.80***	9	155	9.71***

**p = 0.0002.

***p<0.0001.

## Discussion

This study provides evidence for a genetic basis to explain morphological and behavioral differences between inland and coastal populations of swamp sparrows consistent with a local adaptation hypothesis despite the lack of divergence in neutral genetic markers. While maternal effects cannot be completely ruled out, genetic explanations for differences between populations are nonetheless supported and a plastic response to the environment is ruled out. We do not have evidence that any of these traits confer a direct fitness advantage to swamp sparrows. However, strong correlation between environment and suites of traits in closely related species is evidence of similar responses to selection [1]. The repeated biogeographic pattern of larger bills in tidal marsh specialists such as coastal plain swamp sparrows is thought to be an adaptation to new and abundant food resources found in tidal marshes [20], [26]. Salt marshes have the lowest abundance of seeds when compared to other wetlands [27] but very high densities of benthic invertebrates [26]. As a result, salt marsh sparrows tend to consume a larger proportion of invertebrates in their diets then their inland relatives [20]. Further, longer bills might offer increased access to invertebrates hiding in the cracks and crevices of exposed tidal mud. Darker and grayer coloration, a phenomenon common to many vertebrate taxa found in tidal marshes, known as salt marsh melanism, is likely to make sparrows more cryptic in anoxic salt marsh substrates dominated by iron sulfides as opposed to iron oxides [20]. Darker plumage might provide additional protection in humid environments by increasing resistance to feather eating bacteria [28].

Delayed molt, which also has a genetic basis, appears to be an adaptation to allow coastal plain swamp sparrows to undertake more nesting attempts in a longer breeding season. The extended breeding in tidal marsh sparrows is hypothesized to mitigate the observed high nest failure and decrease in clutch size in tidal marsh sparrows, which may also be a response to higher levels of predation [29] in tidal marshes compared to inland marshes [21]. Although, we were unable to directly assess the genetic component of clutch size and breeding season length in laboratory raised birds, the result that molt patterns have diverged in these populations combined with divergence in timing of reproductive status in males is strongly suggestive of genetic element to a divergence in life history. This is one of the few studies to demonstrate that variation in annual rhythm in vertebrate populations at similar latitudes may have a genetic basis.

Lack of detectable genetic divergence in light of evolutionary divergence in swamp sparrows suggests that selection is likely to be strong in tidal marsh populations with adaptation occurring relatively quickly and possibly in the presence of gene flow [20], [23]. Although, it is unknown whether populations are interbreeding, there is a narrow zone of contact between populations making gene flow possible. However, genetic data that are consistent with a gene flow hypothesis are also consistent with a recent divergence hypothesis. Gene trees based on neutral genetic markers may be polyphyletic due to incomplete lineage sorting [13]. In this case, phylogenetic signal is weak because recent separation between populations has not allowed sufficient time for drift and mutation to clear similar ancestral histories between populations [13], [30]. Estimates from divergence in mitochondrial DNA date the most recent common ancestor between subspecies of swamp sparrows to approximately 40,000 years ago with a population expansion occurring more recently at about 10 or 15,000 years ago [23]. Thus, patterns of genetic diversification without phylogenetic signal we find between populations of swamp sparrows are consistent with a mechanism of gene flow in the presence of strong selection, incomplete lineage sorting due to recent divergence or both. In any case, this study provides evidence that ecological adaptation can be relatively fast and strong, perhaps strong enough to precipitate speciation and provides justification for considering morphologically divergent populations as evolutionary important levels of biodiversity.

The results of this study call into question the expectation of reciprocal monophyly of selectively neutral DNA to identify locally adapted subspecies because the stochastic processes responsible for these patterns do not account for the more rapid diversification in genes under selection. There are a growing number of examples of morphological divergence between subspecies in which researchers have been unable to detect divergence in a variety of selectively neutral loci [13]. Recent divergence times are implicated to explain lack of genetic divergence in some cases [30], [31]. In some cases, finding divergence in neutral DNA in morphologically divergent subspecies depends on the type of marker used [32]. Thus, the ability to detect genetic divergence in morphologically distinct populations depends on the time since divergence, the type of genetic markers used and demography. However lack of divergence in neutral DNA between morphologically distinct subspecies has led to the questioning of conservation efforts for such groups [7]. By considering only genetic divergence in neutral DNA to identify subspecies not only do we risk missing an opportunity to understand the dynamic processes of natural selection in action but we also run the risk of losing important contributions to biodiversity. For example, geographically separated populations that exhibit divergence in neutral DNA may not contribute significantly to biodiversity if the populations are biologically indistinct and interchangeable [6]. However, locally adapted populations that exhibit morphological distinctions without detectable divergence in selectively neutral loci are not biologically interchangeable and may have a greater impact on contributing to biodiversity. Local adaptation can happen quickly, but it is not guaranteed. Indeed, it depends on successful colonization followed by mutation on which selection can act. Thus, even if rich environmental gradients are preserved, the loss of locally adapted populations or subspecies may be permanent despite the evolutionary potential of the ecosystem. The findings of this study provide support for the argument that intraspecific morphological variation is an important consideration when accounting for evolutionarily significant levels of biodiversity.

Irrespective of the specific adaptive significance of morphological and behavioral divergence between populations of swamp sparrows, differences in bill size and plumage as well as the timing of reproduction and molt in experimental groups provide evidence of divergence that is the result of local adaptation to different environments rather than to phenotypic plasticity. Because adaptive differences occur in the absence of detectable divergence in neutral markers, we hypothesize that selection on these traits in swamp sparrows is sufficiently strong to override recent divergence and/or ongoing gene flow from inland populations. These results are strongly suggestive of rapid divergence of coastal plain populations with strong selection that is consistent with incipient ecological speciation [16]. That these characters have diverged in similar ways across sparrows of different coastlines and genera suggests that these adaptations solve common problems faced by tidal marsh taxa [1], [20]. The results of this study suggest that discordance in divergence between morphological characters and molecular genetic markers may represent an opportunity to understand ecological mechanisms of incipient speciation. Discordance between morphology and selectively neutral loci has caused controversy in defining subspecies. The results of this study suggest that by ignoring morphological divergence between populations that lack detectable genetic divergence we may be missing an opportunity to conserve evolutionary significant levels of biodiversity and to uncover the evolutionary hot spots that drive adaptive divergence.

## Materials and Methods

We located the nests of breeding swamp sparrows in Woodland Beach Wildlife Area, Delaware (coastal population) and The Glades, Garrett County Maryland (inland population). Nests were monitored until hatching and we collected nestlings when they reached 4 days of age. We collected 17 nestlings (5 nests) from MD and 17 nestlings (6 nests) from DE. We transported nestlings to indoor animal care facilities at the Smithsonian National Zoological Park, Washington, DC where we hand reared them under identical conditions on 12D:12L photoperiod. Nestlings fledged at approximately 10 days and were transferred into group cages. Once nestlings reached independence at approximately 18 days, they were transferred into individual cages (18″L ×9″D ×10 ½″H) where they remained into adulthood on natural photoperiod cycles. All birds were sexed by genetic assignment [33]. We measured body size (tarsus and body mass) and bill length, depth, and width of captives as fledglings and adults using a digital scale (mass) and Tajima calipers. Experimental fledglings were measured between 12–14 days of age. Experimental adults were all measured in the fall of their first year. During the course of the study, we measured various aspects of plumage coloration. Swamp sparrows have complex plumage patterns making it difficult to characterize plumage with one measure. Thus, we focused on areas of the plumage they are often used in the field to differentiate between subspecies. After the first pre-basic molt, we estimated the amount of black on the head and back using digital photography. We measured flank coloration using an Ocean Optics spectrophotometer and estimated the percentage of chestnut feathers in the eye line. After the first pre-alternate molt (which involves only head feathers), we scored crown pattern and estimated the amount of black on the crown and nape. We noted the progression of second basic molt on all birds two times per week beginning on August 26, 2008 and continuing until all birds were deemed finished on October 28, 2008. To confirm reproductive status of molting males, we scored the size of cloacal protuberance relative to full size.

### Rearing diet and conditions

Nestling diet was a mixture of raw lean ground beef, whole grain baby cereal, raw wheat germ, hard boiled egg, carrot, calcium supplement, iron supplement, multi-vitamin supplement, and powdered milk. Nestlings were hand-fed once every half hour until day 10, then once every hour. At day 18, fresh food (see below) was introduced *ad libitum* and hand feeding diet was reduced to once every 3 hours. At, day 24 hand feeding diet ceased. Fresh food diet was provided *ad libitum* along with adult diet (see below) for one week and then fresh food was reduced gradually over the course of the three weeks until birds were on adult diet by approximately day 60. Fresh food diet was a combination of soaked seed, fresh peas, tofu, and egg food. This diet provided birds with a variety of items to choose from while they were becoming independent. Adult diet was *ad libitum* dry seed mixture, 6–8 mealworms every other day and egg food with shell and multi-vitamin once per week. They were provided with grit that contains a calcium supplement.

### Plumage color analyses

For spectrometeric analyses, we analyzed a patch of 8–10 feathers pulled from the flank and affixed to a black background in such a way as to mimic natural arrangement of feathers. We recorded spectral data with an Ocean Optics S2000 spectrometer (range 400–880 nm; Dunedin, Florida) using a micron fiber-optic probe at a 90° angle to the feather surface. Ambient light was excluded with a cylindrical metal sheath attached to the probe tip with the probe held at fixed distance of 6 mm from the feather surface. The reading area was 2-mm diameter of light illuminated with a tungsten-halogen bulb (visible light source). We generated reflectance data relative to a white standard (Labsphere, Inc.). Using spectra acquisition software, OOIBase, we recorded 5 spectra sequentially and averaged the spectra to reduce noise. This process was repeated five times by lifting the probe and replacing it at random locations on the feather sample. An average was taken from the five scans. We quantify flank coloration as the slope of the line of the reflectance spectra in the orange-red portion of the each spectrum (R_575 nm–700 nm_). Digital photographs were taken under identical lighting conditions in the laboratory with a Panasonic 35 mm camera. Digital photographs were analyzed using the masking tool of Corel Paint photo editing software to estimate the amount of black coloration in the back and head. Sections of digital photographs were sampled from the back and head and the masking tool was used to estimate the number of pixels that were black for each section. The amount of black was estimated as the number of black pixels/total number of pixels of the section. Two independent observers performed photo analyses with highly repeatable results (R^2^ = 0.99 head, R^2^ = 0.98 back, n = 11). We estimated the percentage of the eye line that contained chestnut feathers.

After the first alternate molt, we classified rusty cap coloration using scores of 1–4 with 1 having the least rusty coverage and 4 having a full rusty cap [34]. For all males with crown classification 3–4, we measured the length of the rusty cap, black forehead, and black nape on individuals with greater than 50% black on the nape.

### Molt and reproductive condition analyses

We scored primary replacement of post-breeding molt for all 9 primaries counted from proximal to distal as a percentage of the final length. We assigned the following scores: old feathers  =  score 0, missing  =  score 1, below 33% of the length  =  score 2, between 33% and 66% of length  =  score 3, between 66% and less than full grown  =  score 4, full grown new feather  =  score 5. To account for progression of molt, scores of each primary were multiplied by primary number and scores for each primary were summed such that a completed molt would receive a score of 250.

Although males and females were housed separately they were in constant visual and acoustic contact and exhibited signs of breeding condition when on long days. All males sang, and exhibited a full cloacal protuberance. All females laid at least one unfertilized egg during the course of the breeding season and many exhibited behavioral postures associated with sexual receptivity. To confirm reproductive status of molting birds, we scored cloacal protuberance relative to full size. We assigned the following scores: no sign of protuberance  =  score 0, small size  =  score 1, medium size  =  score 2, large or full size  =  score 3.

### Statistical Analyses

We used t-tests to compare individual variables if they were normal. Non-normal data or data with significant heterogeneity in variance transformed using a cube-root transformation (bill size) or an arcsine transformation (percent chestnut in eye-line), or compared with Mann-Whitney U test (crown class). We used a general linear mixed model in SAS [35] to evaluate the differences between subspecies in progression of molt and regression of cloacal protuberance. The molt analysis and cloacal protuberance analysis were conducted separately. The models included either molt score or cloacal protuberance as the dependent variable and subspecies, date and the interaction between subspecies as predictor variables with individual as the repeated measure. Forward step-wise Discriminant Function Analysis (DFA; F to enter  = 1.00) was conducted on the following morphological variables (transformed as described above): Bill volume, tarsus length, percent chestnut in crown, percent black in back, and flank color slope. Wilk's Lamda was used as the test statistic for the significance of the discriminant function. The a posteriori classification was examined for the percent of cases assigned to the correct subspecies and compared to a 50% correct classification based on random classification. The robustness of the ability to classify subspecies based on the DFA was further examined using a cross-validation where the discriminant function was generated from a random sample of half of the individuals and the remaining half was classified. The cross-validation test was conducted on the structural variable (bill size) and plumage-color variable (percent chestnut) that had the highest canonical scores in the total sample DFA.
